# Lifestyle and health behaviour changes of ageing Finnish women during early COVID-19 restrictions

**DOI:** 10.1177/14034948251408592

**Published:** 2026-01-12

**Authors:** Sanni Turunen, Juho Kopra, Saara Lappalainen, Reijo Sund, Heikki Kröger, Toni Rikkonen

**Affiliations:** 1 Kuopio Musculoskeletal Research Unit (KMRU), Institute of Clinical Medicine, University of Eastern Finland (UEF), Kuopio, Finland; 2 School of Computing, University of Eastern Finland (UEF), Kuopio, Finland; 3 Department of Orthopaedics, Traumatology and Hand Surgery, Kuopio University Hospital, Finland

**Keywords:** COVID-19, restrictions, lifestyle, health behaviour, ageing population

## Abstract

**Aims:**

The outbreak of the COVID-19 pandemic and the following lockdown enforced substantial lifestyle changes in people’s everyday lives. This study aimed to assess the impacts of the COVID-19 restrictions on the ageing women’s mental, social and physical well-being.

**Methods:**

Data were collected through two postal questionnaires. Baseline data were obtained in May–June 2019, prior to the pandemic. A follow-up survey was mailed to 760 eligible participants in August–September 2020; 571 women (75.1%) responded, forming the final cohort. The cohort comprised community-dwelling women born between 1932 and 1941 (mean age 82.5 years). Both surveys assessed health, lifestyle, mood, sleep, and technology use, with the follow-up including pandemic-related items.

**Results:**

Dietary habits improved with increased consumption of fruits, vegetables and fish, and decreased intake of sweets, pastries, alcohol and meat. Sleep quality and perceived sufficiency remained stable, although nighttime awakenings declined (*p*=0.023). Social interactions decreased for 43.6% of respondents, yet 20.2% reported receiving more support. Functional capacity deteriorated significantly, with reduced ability to climb several floors, run 100 m and cycle (all *p*<0.001).

**Conclusions:**

**Early lockdown was associated with both positive and negative outcomes. Favourable changes included healthier diets and stable sleep quality. Despite reduced face-to-face contact, perceived social support improved for some participants. However, physical functioning declined, underscoring the need to maintain mobility during prolonged restrictions.**

## Introduction

SARS-CoV-2 (severe acute respiratory syndrome coronavirus 2), also known as COVID-19, had its outbreak at the end of 2019, causing remarkable morbidity and mortality [1]. The risk factors associated with more severe COVID-19 symptoms include ageing, male sex, obesity, hypertension and other medical conditions, such as coronary artery disease, asthma, diabetes and chronic kidney disease [2]. Ageing is one of the main determinants of a serious infection, acute respiratory distress syndrome and death due to the immunosenescence of ageing patients [3,4].

In Finland, the virus started to spread in March 2020, leading to large-scale restrictions implemented by the Finnish government. The restrictions limited activities in several public instances such as swimming halls, libraries, gyms, schools and restaurants. Citizens aged 70 years or older were recommended to avoid social interaction owing to the higher mortality and the lack of vaccination [5].

In a comparison study between 11 countries, Finnish people engaged in the set restrictions more conscientiously than others. Women tended to be more compliant with the restrictions than men, and the regulations were considered to be more effective by older people [6]. Thus, face-to-face contact reduced substantially among Finnish adults in April 2020. The number of face-to-face contacts in the age group of 70–79 years was only half that of younger adults and the overall reduction of contacts in this age group was 88% [7]. Existing studies agree that the closure of public sports venues and limited social contacts reduced physical activity among seniors, who were most affected by these limitations [8,9]. This, in turn, might have had an impact on their psychological well-being as well [9].

The lockdown showed several health ramifications in terms of sleep and dietary patterns. The prevalence of sleeping problems increased widely [10,11]. As to older people, COVID-19-related loneliness and social isolation were associated with a negative impact on sleep [12] but the results remain contradictory: the quality of sleep in a 60+ age group of the Polish and German population was reported to be better than in younger age groups [13]. Interestingly, a Dutch study reported that the changes in sleep depended on the pre-pandemic sleep quality. Those who suffered from insomnia before the lockdown experienced an improvement in sleep quality during the isolation, whereas those who slept well before the pandemic were more likely to suffer from poor sleep quality during the lockdown [14].

In March 2020, the World Health Organization published guidance on how to eat healthily and limit salt, sugar and fat intake as well as avoid alcohol consumption during the self-quarantine [15]. The results of earlier studies, however, show that the guidance was mostly ignored. Food consumption and meal patterns, such as the type of food, were unhealthier during the pandemic. Eating out of control, snacking, and eating more often were also significantly more common [16,17]. In contrast, an Italian study showed a major increase in homemade food consumption while the intake of delivery food decreased moderately. Most participants had not changed the number of meals they ate during the day [18]. Alcohol consumption increased remarkably in the US and Poland [17,19] but decreased moderately in Italy [18].

## Aims

So far, the studies concerning health behavioural changes during the pandemic have mainly focused on the working-age population, while detailed knowledge of seniors and the impacts of the COVID-19 restrictions on them is sparse. This study aimed to explore the changes in the mental, social and physical well-being of ageing women in Finland during early COVID-19 restrictions.

## Materials and methods

### Study design and setting

This study was a repeated-measurements cohort study designed to assess changes in health behaviour, physical functioning and perceived health before and after the COVID-19 pandemic outbreak and related lockdown restrictions. The study was conducted in the North Savo region of Finland as part of the ongoing OSTPRE cohort study [20]. The baseline data were collected between May and June 2019, prior to the COVID-19 pandemic. The follow-up data collection (COVID-19 questionnaire) took place between August and September 2020, approximately five months after the national lockdown restrictions were initiated in March 2020.

### Participants

Eligible participants were women from the OSTPRE cohort who had responded to the baseline questionnaire in 2019. At the time of planning the follow-up, 760 women were invited to participate. A total of 571 returned the follow-up questionnaire, corresponding to a response rate of 75.1%. Only individuals who completed both questionnaires were included in the repeated-measurements analyses. Participants who failed to answer either of the questionnaires or provided incoherent responses were excluded from variable-specific analyses. The proportion of excluded cases per variable ranged between 0.3% and 11.2%.

### Variables and measurements

The main outcomes were self-reported measures across multiple domains of health behaviour, physical ability, sleep and social resources. Continuous variables included age, body weight, body mass index (BMI) and number of medications. Binary variables included use of medication, living alone, having someone to love, and being loved. Ordinal variables included perceived health condition, ambulatory status, ability to climb one or several flights of stairs, ability to walk 0.5 km or run 100 m, ability to ride a bicycle, use of public transport and ability to perform daily activities such as dressing, cutting toenails and hearing speech in a conversation. Sleep-related variables comprised perceived sufficiency and quality of sleep and frequency of waking during the night. Social resource variables covered the frequency of meeting friends or relatives, opportunity to discuss personal matters and perceived adequacy of social support.

Additional variables were collected only at the follow-up (COVID-19 questionnaire), including self-assessed changes in overall health, dietary habits (such as consumption of bread, fish, fruits and berries, meat, milk, pastries, sweets, vegetables and dairy desserts), number of meals and snacks per day, time spent indoors and use of communication or payment technologies (mobile phone, landline, computer, automated teller machine and internet). Perceived effects of COVID-19 restrictions on general health and physical condition were also assessed. All variables were self-reported.

All data were obtained from postal questionnaires administered as part of the OSTPRE cohort. The same format and question wording were used for comparable variables across the two time points to ensure consistency in measurement. New items in the follow-up questionnaire were based on the OSTPRE framework but adapted to capture COVID-19-related lifestyle changes.

### Bias and study size

To minimize potential selection bias, all eligible OSTPRE participants who had completed the baseline questionnaire were invited to the follow-up. Information bias was reduced by using identical questionnaire items for repeated measures. Missing or incoherent responses were excluded for each variable individually to prevent loss of complete cases. The study size was determined by the number of eligible cohort members who had completed both questionnaires.

### Statistical methods

Changes between baseline and follow-up were analysed using paired statistical tests appropriate for the scale of measurement. Continuous variables were compared using the paired-samples *t*-test, binary variables with McNemar’s test, and ordinal variables with the Sign test. Variables measured only at follow-up were summarized descriptively and by visual inspection of Likert-scale data. Missing data were handled through variable-specific exclusion without imputation. All analyses were performed using IBM SPSS Statistics, version 27. Data preprocessing, quality checks and figures were conducted in R, version 4.3.1.

## Results

Overall, self-perceived health improved during the early COVID-19 restrictions. In the COVID-19 questionnaire, 17.7% (*n*=100) of women gave a more positive assessment of their health compared with the time before the pandemic outbreak, while only 12.7% (*n*=72) reported their health as deteriorated (*p* = 0.040). A slight decrease in the mean BMI was seen during the follow-up. In the baseline questionnaire the mean BMI was 27.2 (SD 4.8) while in the COVID-19 questionnaire it was 27.0 (SD 4.8) (*p* < 0.001). The proportion of respondents using medication showed a modest increase from 94.9% to 96.6% (*p* = 0.039). The average number of medications taken changed from 4.7 (SD 2.9) to 5.2 (SD 3.2). Table I provides the self-reported characteristics of the study population.

**Table I.** Changes in self-reported characteristics of the respondents.

	Baseline Mean (SD)	COVID-19 follow-up Mean (SD)	*p*
**Age, years**	81.3 (2.7)	82.5 (2.7)	
**Body weight, kg**	68.6 (12.8)	68.1 (12.8)	<0.001^a^
**Body mass index, kg/m** ^2^	27.2 (4.8)	27.0 (4.8)	<0.001^a^
**Number of medications**	4.7 (2.9)	5.2 (3.2)	<0.001^a^
	**Baseline** % (*n*)	**COVID-19 follow-up** % (*n*)	***p***
**Health condition**			0.040^b^
Poor or very poor	7.6 (43)	7.6 (43)	
Average	53.1 (301)	47.9 (272)	
Good	34.4 (195)	39.3 (223)	
Very good	4.9 (28)	5.3 (30)	
**Use of medication**			0.039^c^
No	5.1 (28)	3.4 (19)	
Yes	94.9 (519)	96.6 (537)	

Statistical test used: ^a^paired sample *t*-test, ^b^sign test, ^c^McNemar test.

No significant differences were observed in overall ambulatory status (*p* = 0.663). Enquiring about the specific forms of performance, however, some changes in assessed variables were observed. Up to 9.5% (*n*=50) of the respondents reported deterioration in their ability to walk up one floor (*p* = 0.012) and as many as one-fifth of the participants (20.3%, *n*=103) experienced more difficulty in walking up several floors (*p* < 0.001). Similarly, 11.4% (*n*=57) of women assessed their ability to ride a bicycle as worse than before (*p* < 0.001) and 15.9% (*n*=83) of them reported a negative change in their ability to run 100 m (*p* < 0.001). In the baseline, 62.8% (*n*=338) were completely incapable of running 100 m, while in the COVID-19 questionnaire, as many as two-thirds (66.6%, *n*=363) of women reported the same. Up to 13% (*n*=68) of women reported reduced capability of travelling by bus or train (*p* < 0.001). The incapability of cutting toenails increased from 10.9% to 14.3% (*p* = 0.024). Table II reports self-perceived changes in the physical capacity and the capability of carrying out everyday tasks before and after the COVID-19 outbreak.

**Table II.** Self-perceived changes in the physical capacity and the capability of carrying out everyday tasks before and after the COVID-19 outbreak.

	Baseline % (*n*)	COVID-19 follow-up % (*n*)	*p*
**Ambulatory status**			0.663^a^
Disabled	0.8 (4)	0.4 (2)	
Limited, less than 1 km	20.5 (107)	21.5 (112)	
Full ambulatory, not able to run	47.4 (247)	46.5 (242)	
Full, able to run	31.3 (163)	31.5 (164)	
**Able to go up one floor**			0.012^a^
No	6.5 (35)	9.0 (49)	
Yes, with difficulty	10.6 (57)	12.6 (69)	
Yes	82.9 (446)	78.4 (428)	
**Able to go up several floors**			<0.001^a^
No	24.4 (131)	29.9 (159)	
Yes, with difficulty	22.0 (118)	25.2 (134)	
Yes	53.6 (288)	44.8 (238)	
**Able to walk 0.5 km without taking a break**			0.302^a^
No	15.3 (83)	16.0 (87)	
Yes, with difficulty	9.9 (54)	10.5 (57)	
Yes	74.8 (406)	73.6 (401)	
**Able to run 100 m**			<0.001^a^
No	62.8 (338)	66.6 (363)	
Yes, with difficulty	19.0 (103)	17.1 (93)	
Yes	18.6 (101)	16.3 (89)	
**Able to ride a bicycle**			<0.001^a^
No	59.7 (314)	64.3 (342)	
yes, with difficulty	8.6 (45)	9.2 (49)	
Yes	31.7 (167)	26.5 (141)	
**Able to travel by bus or train**			<0.001^a^
No	6.4 (35)	12.1 (66)	
Yes, with difficulty	4.8 (26)	9.2 (50)	
Yes	88.8 (485)	78.7 (429)	
**Able to read a newspaper**			0.728^a^
No	3.6 (20)	2.9 (16)	
Yes, with difficulty	3.6 (20)	3.0 (17)	
Yes	92.8 (519)	94.1 (528)	
**Able to get dressed without help**			0.629^b^
Difficultly or not at all	2.8 (16)	2.3 (13)	
Yes	97.2 (548)	97.7 (552)	
**Able to cut toenails**			0.024^a^
No	10.9 (61)	14.3 (80)	
Yes, with difficulty	14.4 (81)	14.4 (81)	
Yes	74.7 (419)	71.3 (400)	
**Able to hear speech in a conversation**			0.980^b^
Difficultly or not at all	9.0 (50)	10.7 (60)	
Yes	91.1 (505)	89.3 (503)	

Statistical test used: ^a^sign test, ^b^McNemar test.

The quality of sleep (*p* = 0.425) and self-perceived sleep sufficiency (*p* = 0.769) remained similar in both questionnaires. In contrast, the frequency of waking up at night decreased (*p* = 0.023). The proportion of participants who seldom or hardly ever wake up at night increased from 15.5% (*n*=86) to 19.9% (*n*=111). Altogether, 22.3% (*n*=123) of the respondents woke up less frequently than before. Table III shows the changes in sleep before and after the COVID-19 outbreak.

**Table III.** Changes in sleep before and after the COVID-19 outbreak.

	Baseline % (*n*)	COVID-19 follow-up % (*n*)	*p*
**Sufficient sleep**			0.769^a^
Seldom or hardly ever	12.5 (66)	12.6 (64)	
Often	39.7 (210)	35.5 (179)	
Almost always	47.8 (253)	52.1 (264)	
**Quality of sleep**			0.425^a^
Poor	3.1 (17)	3.9 (21)	
Relatively poor	18.0 (99)	16.7 (90)	
Relatively good	61.7 (340)	62.2 (335)	
Good	17.2 (95)	17.3 (93)	
**Frequency of waking up at night**			0.023^a^
Seldom or hardly ever	15.5 (86)	19.9 (111)	
Sometimes	40.3 (227)	37.5 (209)	
Often or very often	44.4 (250)	42.7 (238)	

Statistical test used: ^a^sign test.

A minor increase in the proportion of individuals living alone was perceived during the following period (*p* < 0.001). In the baseline questionnaire, 56.8% (*n*=323) reported living alone, while in the COVID-19 questionnaire the percentage had increased to 60.2% (*n*=342). The greatest change was discovered in meeting other people (*p* < 0.001): as many as 43.6% (*n*=230) of the respondents reported meeting their friends and relatives less frequently than before. The proportion of women meeting their closest friends less than once a month increased from 5.9% (*n*=32) to 10.0% (*n*=56). In turn, as many as 15.0% (*n*=79) of women reported meeting their friends and relatives more often than before. Just over one-fifth (20.2%, *n*=102) reported receiving more support whereas only 10.1% (*n*=51) experienced receiving less support than before the pandemic (*p* < 0.001). The social resources of the study population before and after the COVID-19 outbreak are shown in Table IV.

**Table IV.** Social resources of the study population before and after the COVID-19 outbreak.

	Baseline % (*n*)	COVID-19 follow-up % (*n*)	*p*
**Living alone**			<0.001^a^
No	43.2 (246)	39.8 (226)	
Yes	56.8 (323)	60.2 (342)	
**Having someone that loves you**			0.103^a^
Yes	11.3 (56)	12.4 (66)	
No	88.7 (441)	87.6 (468)	
**Having someone to love**			0.781^a^
Yes	8.8 (86)	10.9 (59)	
No	91.2 (456)	89.1 (481)	
**Frequency of meeting friends and relatives**			<0.001^b^
Hardly ever or less than once a month	5.9 (32)	10.0 (56)	
1–3 times a month	10.0 (54)	14.0 (78)	
Once a week	21.0 (113)	32.1 (179)	
2–3 times a week	27.1 (146)	27.1 (151)	
More than three times a week	36.0 (194)	16.8 (94)	
**Having a chance to discuss with someone if necessary**			0.253^b^
Rarely or never	5.2 (29)	7.1 (40)	
Occasionally	23.7 (133)	16.8 (95)	
Often	31.7 (178)	35.3 (200)	
Always	39.4 (221)	40.8 (231)	
**Having enough support**			<0.001^b^
Not enough/no friends or relatives	4.3 (22)	4.2 (24)	
Somewhat enough	44.5 (227)	33.6 (190)	
Fully enough	51.2 (261)	62.2 (352)	

Statistical test used: ^a^McNemar test, ^b^sign test.

**Figure 1.**
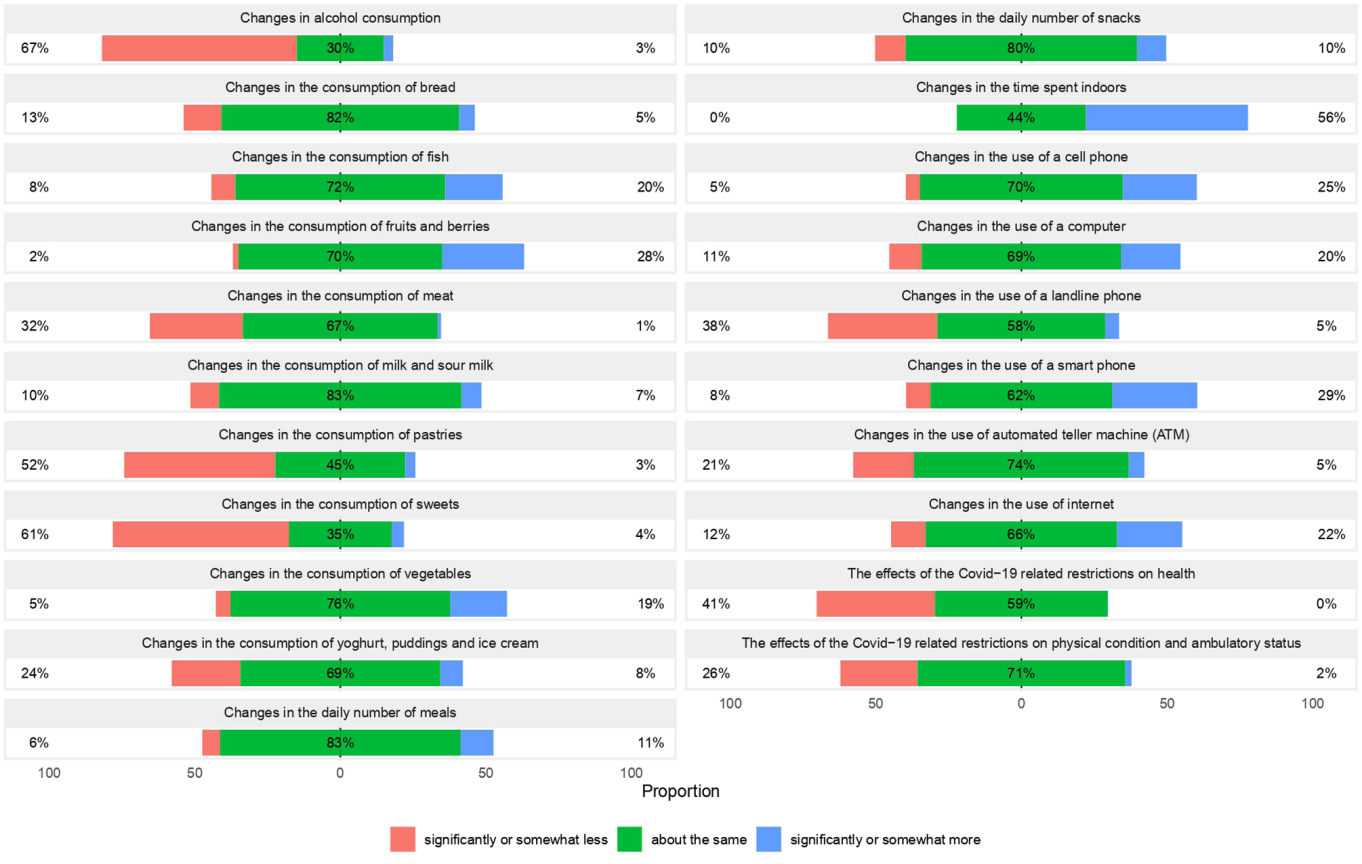
Self-assessed lifestyle changes during the COVID-19 restrictions.

In general, the number of daily meals and snacks did not change much after the pandemic outbreak. More prominent changes were discovered in consuming different food products: almost two-thirds (61%) reported eating fewer sweets and approximately half of the participants (52%) reported eating fewer pastries than before. The consumption of meat had likewise decreased: 32% reported eating less meat than before. On the contrary, 20% of participants had eaten more fish, 19% had added more vegetables to their diet and as many as 28% had consumed more fruits and berries. The majority (67%) of the respondents had reduced their alcohol consumption, while only 3% reported drinking more alcohol. These self-assessed lifestyle changes during the early COVID-19 restrictions are detailed in Figure 1. In addition, 9% of women had quit smoking (data not shown).

More than half (56%) of women reported spending more time indoors because of the restrictions and nearly as many (49%) reported having fewer leisure activities than before. On the contrary, the use of smartphones, cell phones, computers and the internet increased (Figure 1). A small proportion (7%) of women had cancelled their doctor appointments because of the ongoing pandemic (data not shown). Interestingly, 41% of women perceived a negative impact of COVID-19 restrictions on their health condition (Figure 1), while in repeated questions (Table I) the outcome was rather the opposite. Similarly, just over one-quarter (26%) reported their ambulatory status as deteriorated (Figure 1) but, as with changes in health, there was no significant difference in repeated questions (Table II).

## Discussion

In this study, we analysed changes in the lifestyle and health behaviour of a sample population of ageing Finnish women during the early COVID-19 restrictions. The results indicate both negative and positive changes in the lives of the study population. Overall, the health behaviour changes shifted towards positive. The food choices during the initial phase of the pandemic were healthier than before, the changes in the sleep patterns were beneficial as well, there was a slight decrease in BMI and the self-perceived health was better than before. The time spent on smartphones, cell phones, computers and the internet increased. Even though the seniors had fewer face-to-face contacts, the proportion of seniors receiving support increased in the beginning of the lockdown. However, the result suggests that there was a slight impairment in older women’s physical performance during the early COVID-19 restrictions.

The main limitation of our study was the use of self-reported data, some of which were available only in the COVID-19 questionnaire. Thus, not all the questions were repeated, which might affect the reliability of the findings. It is also important to consider that ageing itself is associated with physiological, social and behavioural changes. Thus, while the study population consisted of ageing women, some of the changes might be associated with common ageing-related alterations, which are difficult to estimate. In addition, this study examined changes only during the initial phase of the pandemic, so the results might have been different if the follow-up period had been longer. The strength of our study was the relatively short time interval between the questionnaires. This reduces the probability of recall bias, especially in the questions that were included only in the COVID-19 questionnaire and addressed changes resulting from the early stages of the pandemic. In addition, most of the health questions were enquired not only after the outbreak of the pandemic but also a year prior. Therefore, the repeated questions increase the reliability of our observations.

The effect of the COVID-19 pandemic on women’s ambulatory status is difficult to differentiate from biological ageing. The deterioration in women’s ability to run, walk up the stairs and ride a bicycle might stem from ageing-related impairment but also from spending more time indoors and having a lower engagement in normal leisure activities during the pandemic. Altogether, ageing might be the most natural cause of the changes observed. Yet, the link between inactivity and decreased physical function is widely demonstrated in existing studies. The prevalence of frailty in older adults with an inactive lifestyle and sedentary behaviour is generally higher compared with those with sufficient levels of physical activity. This might expose the seniors to unfavourable health factors, such as frailty, falls and dependence on other people [21]. Consequently, it is important to consider alternative ways to maintain an active lifestyle during a global pandemic and to bring these risks to the attention of the older population.

The results of this study show that the eating behaviour was in line with the World Health Organization recommendations [15]. The diet changes were mainly positive, with increased consumption of fruits, vegetables and fish and decreased consumption of sweets, pastries, alcohol and meat. At the same time, there were no notable changes in the number of meals or snacks. The explanation for this might be the changes in grocery shopping in times of pandemic: the seniors might have received more help from others, which, in turn, has helped them to maintain a more balanced diet.

As for the sleeping patterns during the early pandemic, the quality of sleep and self-assessed sleep sufficiency did not change notably in our sample population. Instead, the frequency of waking up in the middle of the night decreased. During the COVID-19 pandemic, older age has been suggested as a protective factor for sleeping difficulties [22], which might be explained by the lifestyle of older people. Since our sample population consisted of pensioners the pandemic might not have changed their lives as radically as it did the life of the working-age population.

Although the changes in working life did not affect our study cohort, a significant part of the respondents reported meeting their friends and relatives less frequently than before the restrictions. This trend is consistent with existing studies showing that old people were exposed to loneliness during the COVID-19 pandemic [7,23]. In addition to the nationwide recommendations of COVID-19 isolation, the limited access to social events and public places probably contributed to the results. However, regardless of fewer physical contacts, one-fifth of our participants reported receiving more support during the initial phase of the lockdown than before it. At the same time, the use of smartphones, cell phones, computers and the internet increased, which presumably has helped the seniors to stay in contact with their community and family members. In fact, the World Health Organization has recommended maintaining social networks via electronic devices if physical social contacts are limited [24]. This implies how seniors should be encouraged to use digital technology to counteract loneliness in similar conditions.

Although a small proportion of women assessed their subjective health as deteriorating, the repeated questions show that the difference was negligible. In the early stages of the COVID-19 restrictions, a larger proportion of women regarded their health condition as good or very good compared with the corresponding results before the beginning of the pandemic. Similar findings have also been reported among Swedish seniors [23]. A possible explanation for this is the higher score of life satisfaction and lower level of social exclusion of older adults in Nordic countries compared with the other European nations. In Nordic countries, existing material resources and available basic services might contribute to happiness when social interactions are limited [25]. To confirm, the utilization of health and social services among our study attendees was not limited by the COVID-19 infection and there was no change in the basic services they received during the restrictions.

## Conclusion

The COVID-19 restrictions might not have had as significant a negative change in the health of ageing adults as was previously conceived. The time spent indoors increased and women had fewer face-to-face contacts. However, they adopted new ways to keep in touch with their friends and relatives, while receiving adequate support. The dietary changes during the early pandemic were also healthier. However, the restrictions seem to have introduced some deterioration in ageing women’s physical capability.
